# Variability in cognitive strategies in a numerical discrimination task: individual and comparative insights from day-old domestic chicks (*Gallus gallus*)

**DOI:** 10.1093/jas/skaf351

**Published:** 2025-10-10

**Authors:** Kimberly Brosche, Lucia Regolin, Agnese Zazio, Rosa Rugani

**Affiliations:** Comparative Cognition Lab, Department of General Psychology, University of Padua, Padova 35131; Comparative Cognition Lab, Department of General Psychology, University of Padua, Padova 35131; Comparative Cognition Lab, Department of General Psychology, University of Padua, Padova 35131; Comparative Cognition Lab, Department of General Psychology, University of Padua, Padova 35131

**Keywords:** absolute numerosity, domestic chick, Gallus gallus, individual strategy, numerical discrimination, relative numerosity

## Abstract

Sensitivity to numbers is a crucial cognitive ability. Numerical discrimination, defined as the ability to distinguish between different numerosities, allows animals to reduce predation risk, increase foraging efficiency, and maximize food intake, and has been documented in primates, amphibians, fish, birds, and insects. Evidence from day-old chicks suggests that basic numerical competencies are part of a precociously available cognitive toolkit that guides animals in their interaction with the environment. To discriminate between two numerosities, animals could employ either an absolute or a relative strategy. Absolute discrimination requires identifying a specific numerosity (e.g., 10) against others. Relative discrimination consists in selecting the higher (or the lower) numerosity in any pair. Which strategy is preferred differs across species: Humans and fish preferentially adopt a relative strategy, while honeybees apply an absolute strategy. This posed the question how strategy preferences evolve phylogenetically and develop ontogenetically. This study explores whether 3-d-old chicks (*Gallus gallus*) spontaneously adopt an absolute or relative strategy. During rearing, 124 chicks were exposed to a numerical comparison (5 vs. 10; experiment 1) and learned that one numerosity (10) was associated with food. During testing, which consisted of a 6-min unrewarded observation, each chick was presented with the numerosity associated with food (10, the choice of which would indicate an absolute strategy) alongside an unfamiliar numerosity consistent with the relative strategy (20, which is larger than 10). Based on previous evidence, we hypothesized that chicks would spend more time near the numerosity corresponding to their preferred strategy. Chicks did not show a significant preference on a group level, regardless of whether the higher (experiments 1 and 2) or the lower numerosity (experiment 3) had been associated with food during rearing. Interestingly, chicks exhibited significant individual preferences for one or the other strategy that cannot be explained by the novelty of the stimuli (experiment 4) or by the use of physical variables (experiment 2). These findings suggest either that diversity of numerical discrimination strategies was evolutionary favorable in domestic chickens’ natural environments, or that, at this early stage of ontogeny, young chicks’ strategies are still highly variable and flexible.

## Teaser

Adding to previous research that has demonstrated that day-old chicks possess various numerical abilities, this study highlights significant individual variability in the strategies chicks employ to discriminate between numerosities. Individual variability in numerical discrimination strategies observed in chicks may reflect evolutionary adaptability or early ontogenetic flexibility in cognitive development.

## Introduction

Numerical abilities play a crucial role in animals’ survival, as animals frequently face quantitative choices in their natural environment. Numerical discrimination, the capability to discern between two numerosities and evaluate which is smaller or larger, is essential for making adaptive decisions (Vallortigara et al., 2010a; Nieder, 2020a). For example, according to the optimum foraging theory, animals should prefer more abundant food sources (Krebs, 1974). In a similar vein, animals are required to choose the optimal group size in various contexts, depending on within-group resource competition, the need for cooperation, or predation pressure against which larger groups provide better protection (Alexander, 1974; Krebs et al., 2012).

Given its adaptive significance, numerical discrimination has been widely observed across diverse animal species in both natural and seminatural settings using ecologically relevant stimuli (cf Agrillo and Bisazza, 2014). For example, angelfish (*Pterophyllum scalare*) (Gómez-Laplaza et al., 2019), horses (*Equus caballus*) (Uller and Lewis, 2009), and pigs (*Sus scrofa domesticus*) (Gustafsson et al., 1999; Uller, 2009) choose the larger quantity when presented with 2 sets of food items. Analogously, swordtails (Buckingham et al., 2007), minnows (Hager and Helfman, 1991), and baboons (Piantadosi and Cantlon, 2017) select the larger group of conspecifics. Remarkably, these numerical abilities can be observed at an early stage of development, in the absence of any numerical training, as demonstrated in day-old domestic chicks. Chicks approach the larger group of artificial social companions (bidimensional squares reared with them shortly after birth), even when object size is manipulated to rule out the possible use of quantitative cues such as overall area or perimeter (Rugani et al., 2010; Rugani et al., 2020).

More controlled laboratory studies have complemented spontaneous-choice experiments, by training animals to discriminate between numerosities represented by arrays containing varying numbers of items, rather than inherently reinforcing stimuli (e.g., food or conspecifics). For example, young domestic chicks (Rugani et al., 2008), pigeons (Honig and Stewart, 1989; Diaz and Wasserman, 2023), and primates (Brannon and Terrace, 1998; Cantlon and Brannon, 2007) have been trained to compare, order, or select arrays based on numerosity, even when continuous variables (quantitative cues that covary with numerosity, such as overall perimeter, area, or volume) were controlled for. This evidence illustrates that numerosity is a salient property that nonhuman animals can perceive and use in decision-making. However, it remains unclear which strategy animals learn and subsequently apply in numerical discrimination.

To succeed in numerical discrimination, animals could learn and subsequently apply either an absolute or a relative strategy. In learning to discriminate between 5 and 10, with 10 being the rewarded numerosity, an animal can use either an absolute strategy, selecting 10 regardless of the alternative numerosity, or a relative strategy, consistently choosing the larger numerosity, irrespective of the specific numerosities. Using a relative rule (Davis and Pérusse, 1988) is sufficient in most natural contexts, such as foraging and group size choice, where the consistent selection of the larger amount of food or conspecifics is usually adaptive. Nevertheless, whether this strategy is more or less cognitively demanding is debated. Nieder (2020b) posits that the relative strategy is cognitively less demanding than the absolute strategy, as it does not require representations of precise cardinal values. Others uphold the contrary: to make a relative judgment, animals need to process not just one but both numerosities, compare them, and finally derive an abstract rule (Thomas, 1980; Pepperberg and Brezinsky, 1991).

Previous research has demonstrated that animals are able to learn numerical tasks successfully, applying either strategy. For example, Davis (1984) successfully trained a raccoon to select arrays of three objects out of various numerosities. Conversely, applying a relative rule, a gray parrot (Pepperberg and Brezinsky, 1991) and horses (Hanggi, 2003) succeeded in indicating the larger or smaller array. Even though animals can be trained to solve numerical tasks using either rule, only a handful of studies have directly assessed whether animals employ an absolute or a relative strategy to solve numerical discrimination.

Preference for either strategy, at the individual or species level, can be probed by first training animals on a specific numerical comparison, then testing them with a different comparison where the two strategies yield conflicting predictions. More precisely, animals can be trained to receive a reward for selecting 10 when presented with the comparison 5 vs. 10. Subsequently, at test, 10, which was the previously reinforced stimulus, is presented alongside a novel and larger numerosity, for example, 20, congruent with the relative rule. If animals have learned, during training, to solve the task by applying an absolute rule “choose 10”, they are expected to still select 10 in a novel comparison 10 vs. 20, regardless of whether the second numerosity is smaller or larger than 10. If, on the other hand, they have learned a relative rule during training “always select the larger numerosity”, they should choose 20. Such a procedure has hitherto been implemented to study fish (Miletto Petrazzini et al., 2015; Miletto Petrazzini et al., 2016), humans (Miletto Petrazzini et al., 2016), and honeybees (Bortot et al., 2019).

Interestingly, the three species differed in the employed strategy. Angelfish (*P. scalare*) and humans applied a relative strategy when presented with the numerosity pairs 5 vs. 10 and 10 vs. 20 (Miletto Petrazzini et al., 2016). That is, instead of choosing the numerosity that was directly reinforced during training, for example, 10, they selected the unfamiliar numerosity based on its relative magnitude, either smaller or larger, rather than the absolute numerosity reinforced during training. Similarly, guppies (*Poecilia reticulata*) used the relative strategy when trained with 6 vs. 12 and tested with 3 vs. 6 or 12 vs. 24 (Miletto Petrazzini et al., 2015). However, when specifically trained to consistently select numerosity 4, guppies also demonstrated the ability to follow an absolute strategy (Miletto Petrazzini et al., 2015). In contrast to humans and fish, honeybees adopted an absolute strategy when discriminating 3 from 2 or 4 (Bortot et al., 2019). Bees were trained to associate the numerosity 3 with rewards, either distinguishing it from a smaller (2) or a larger (4) numerosity. During testing, they consistently chose 3 over unfamiliar numerosities (respectively 4 or 2), consistent with an absolute strategy. Given these contrasting results, it is highly informative to extend the assessment of strategy preference in numerical comparison to hitherto untested species.

One promising species to provide further insights into the mechanisms underlying strategy preferences is the domestic chick (*Gallus gallus*): a precocial bird that possesses a range of cognitive abilities soon after birth (Vallortigara et al., 2010b). Probing the preference of another vertebrate clade, birds, allows us to understand whether a preference for relative strategies might be shared among all vertebrates, or whether it is limited to mammals (humans) and fish (angelfish and guppies). Notably, previous research has revealed remarkable numerical abilities in young domestic chicks, ranging from numerical discrimination (Rugani et al., 2008; Rugani et al., 2010; Rugani et al., 2013a; Rugani et al., 2020) ordinality (Rugani et al., 2007; Rugani and Regolin, 2020; Rugani and Regolin, 2021) to arithmetic (Rugani et al., 2009) and proportional reasoning (Rugani et al., 2014a; Rugani et al., 2016). In particular, chicks proved able to select the group containing the larger number of *familiar* imprinting objects (e.g., yellow capsules chicks experienced during rearing) even when the overall numerosity of the two groups was identical due to the presence of unfamiliar objects; for example, pink capsules (Rugani et al., 2010). This indicates that chicks initially discern between familiar and unfamiliar objects, and subsequently estimate the numerosity of each group to select the one containing the larger number of familiar objects (Rugani et al., 2010). Nevertheless, when, in the same study, a different group of birds was initially reared with a group of objects and then tested with new objects that differed in color, size, and shape with respect to the imprinting ones, chicks approached the familiar numerosity rather than the larger one (Rugani et al., 2010). Overall, this evidence indicates that chicks apply either relative or absolute strategies depending on the context. In a study aimed at investigating sensitivity to size illusions (Rosa Salva et al., 2013), chicks during rearing learned to discriminate between two differently-sized circles and to associate one of them with food: for some chicks, it was the smaller circle, and for others, the larger one. In the subsequent test, chicks’ susceptibility to a visual illusion was probed. Chicks exhibited a preference for the apparent relative size (the circle appeared larger or smaller depending on the distractors surrounding it) congruent with the reinforcement during rearing. Using an experimental design similar to the one employed in the present research (Rugani et al., 2013b; Rugani et al., 2014b), it has been shown that chicks spent more time close to a panel depicting a numerosity (e.g., 10) that had been associated with food during rearing, compared to a panel depicting a numerosity that had not been associated with food (e.g., 5). Although these latter studies demonstrated chicks’ ability to perform numerical discrimination, they could not pinpoint which strategy chicks utilized to correctly select the numerosity they had associated with food, as the birds were tested with the same two numerosities experienced during rearing. Therefore, the strategy employed by chicks to solve numerical discrimination tasks remains unknown.

The present study aims to address this research gap, by conducting four experiments with young domestic chicks. In experiment 1, chicks learned to associate food with 10 (positive stimulus, *S*_p_) and not with 5 (neutral stimulus, *S*_n_) homogenous black squares. At test, they faced 10 vs. 20 identical squares. Here, 10 (the *S*_p_), was consistent with the absolute strategy, while 20 was the stimulus corresponding to the relative strategy. In experiment 2, we aimed to assess whether chicks’ preferences depend on physical variables. To this aim, during rearing and testing, we used the same numerosities as in experiment 1, employing heterogeneous stimuli successfully used in previous studies, consisting of elements varying in shape, color, and size (Rugani et al., 2013b; Rugani et al., 2015). Experiment 3 assessed whether chicks would show the same preference if the smaller, rather than the larger, numerosity was reinforced during rearing. For this purpose, chicks learned to associate 10 (*S*_p_), rather than 20 (*S*_n_), with food and were subsequently tested on 5 vs. 10. In all these experiments, a potential preference for the relative strategy could be attributed to a general preference for novel stimuli, as the stimulus corresponding to the relative strategy necessarily needs to be unfamiliar. To rule out this alternative explanation, in experiment 4, chicks learned to associate food with 20 (*S*_p_), rather than 10 (*S*_n_). Differently from the previous three experiments, no choice corresponding to the absolute strategy was offered at test, as the *S*_p_ (20) was absent. Chicks could choose between 10 (a familiar and larger numerosity corresponding to the relative strategy but never associated with food during rearing) and 5 (unfamiliar numerosity, incongruent with the relative strategy, *S*_u_).

**Figure 1.**
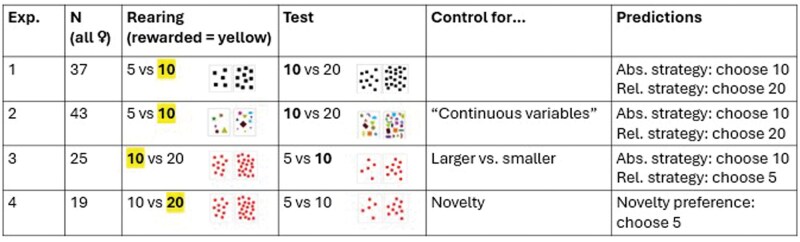
Overview of the 4 experiments. In the columns, we report the sample size (*n*), stimuli used during rearing and in the test (with the reinforced numerosity in bold), what each experiment controlled for, and the predictions made. Abs. = correct choice absolute strategy, Rel. = correct choice relative strategy, Novel = correct choice in case of a preference for novelty.

We hypothesized that, if chicks spontaneously adopt an absolute strategy, they should spend more time close to the numerosity associated with food during rearing (i.e., 10 in experiments 1, 2, and 3). However, if they prefer a relative strategy, they should select whichever numerosity is larger (experiments 1 and 2) or smaller (experiment 3) depending on the food-numerosity association established during rearing. In addition, if chicks’ strategy is independent from physical variables, their preference should not differ between experiments 1 and 2. Experiments 1–3 do not allow to disentangle between a preference for the relative strategy and a general preference for novelty; this was the aim of experiment 4. If chicks do not show a significant preference for the unfamiliar stimulus in experiment 4, we can rule out that their potential preference for the relative strategy in previous experiments was solely due to a general preference for unfamiliar stimuli. The experiments, the corresponding numerosities and the predictions are summarized in Figure 1.

In addition to, or instead of, a group-level strategy preference, individual preferences may emerge. Specifically, some chicks may prefer the stimulus corresponding to the absolute strategy and others that corresponding to the relative strategy, in all experiments but the novelty control (experiment 4).

## Materials and Methods

We conducted four experiments with four separate groups of chicks. Housing conditions, rearing, and testing procedures were identical for all groups and are hence described jointly.

### Institutional animal care and use committee statement

We conducted the research in accordance with the European Union Directive in place at the time the study was conducted (86/609/CEE) on the protection of animals used for scientific purposes, which mandates the implementation of the Three Rs principles (Replacement, Reduction, and Refinement) (Committee for Medicinal Products for Human Use (CHMP) and Committee for Medicinal Products for Veterinary Use (CVMP), 2016) and sets minimum standards for housing, care, and use of animals.

Additionally, we adhered to the ARRIVE guidelines (Animals in Research: Reporting In Vivo Experiments) (Percie Du Sert et al., 2020) to ensure transparent and comprehensive reporting of animal research.

The experiments were approved by the Italian Ministry of Health (permit number: 5/2012 B emitted on 10 January 2012). All procedures employed in these experiments were examined and approved by the Ethical Committee of the University of Padua (Comitato Etico di Ateneo per la Sperimentazione Animale—C.E.A.S.A.) as well as by the Italian National Institute of Health (N.I.H.).

The study was based on behavioral observation and positive reinforcement only. The animals in the study were hence never subjected to invasive or harmful procedures.

### Subjects and housing

Subjects were female “Hybro” domestic chicks (*G. gallus*), a local variety of the White Leghorn breed (for sample sizes, see Figure 1). As in previous studies applying a similar procedure (e.g., Regolin et al., 2011; Rosa Salva et al., 2013; Rugani et al., 2013b), we decided to only include female chicks as they respond better to the procedure employed, which was based on precocial experience (cf. Vallortigara et al., 1990; Regolin et al., 2005). Thirty-seven chicks participated in experiment 1, 43 in experiment 2, 25 in experiment 3, and 19 in experiment 4. Chicks were randomly assigned to the experiments.

Chicks were obtained every Monday, at a few hours of age, from as local commercial hatchery (Agricola Berica, Montegalda, Vicenza, Italy), where they had already been sexed. On arrival, the chicks were housed individually in standard metal cages (28 × 32 × 40 cm). The rearing room was constantly monitored for temperature (28 to 31 °C) and humidity (68%) and was illuminated continuously by fluorescent lamps (36 W) located 45 cm above each cage. Water, placed in transparent glass jars (5 cm in diameter, 5 cm high) centrally on one of the long sides of the cage, was available *ad libitum*. The position of the water jar was shown to chicks the first day by gently dipping their beak in water. Food (standard chick starter crumbles) was also available *ad libitum*, behind the panels depicting the stimuli. An artificial imprinting object (a red capsule measuring 2 × 3 cm) was suspended (approximately at the chick’s eye level) in each rearing cage to alleviate social isolation (Rugani et al., 2013b).

After the test, chicks were kept in groups of two or three and, at the end of the week, they were donated to local farmers.

### Exposure to the stimuli during rearing

We employed a procedure that has previously proven successful in testing numerical discrimination in young chicks (Rugani et al., 2013b; Rugani et al., 2014b). During the first 2 d, the “rearing period” (Monday and Tuesday), four screens (10 × 14 cm, with a base of 10 × 10 cm for the food jar), positioned about 10 cm in front of each corner of the cage, were simultaneously present in each chick’s cage. All screens were covered with static 2D images on the front and the back (each printed on 5 × 6 cm cardboard sheets) depicting a certain number of elements. Two screens depicted identical pictures of the Positive Stimulus (*S*_p_, the numerosity associated with food) and two depicted identical pictures of the Neutral Stimulus (*S*_n_, the numerosity not associated with food). During the rearing period, the chicks found food (jars with crumbles) behind the screens with the Positive Stimulus (*S*_p_), while the two screens depicting the Neutral Stimulus (*S*_n_) hid an empty jar; for an overview of the numerosities constituting *S*_p_ and *S*_n_ in each experiment, see Figure 1. To prevent the chicks from learning to identify the stimuli on the basis of the configuration of the items, six different pairs of stimuli were used. Moreover, to avoid positional learning, every time the stimuli were replaced, the screens were also rotated from corner to corner. On the 2 d of exposure (Monday and Tuesday; first 2 d of life), the stimuli were changed and rotated three times a day.

During the penultimate (fifth) exposure period, the food jars behind the panels depicting the positive stimuli contained only a reduced amount of food (approximately one-third of the previous amount). This was further reduced during the last exposure period (to approximately a fifth of the maximum amount). This served to increase chicks’ motivation during the test (Regolin et al., 2005) without compromising the association between food (even if reduced in quantity) and the positive stimulus.

### Stimuli

The stimuli consisted of a predefined number of homogenous (black or red squares) or heterogeneous (various dimensions, shapes, and colors) elements. The distance between each element and the borders of the cardboard was at least 0.5 cm and the interelement distance ranged between 0.3 cm and 3.8 cm.

The squares constituting the homogenous stimuli (black in experiment 1, red in experiments 3 and 4) had a side length of 1 cm each. The color red was chosen for experiments 3 and 4 to enhance the attractivity of the stimuli to the chicks (Taylor et al., 1969; Salzen et al., 1971; Ham and Osorio, 2007; Santolin et al., 2020). To reduce the relevance and reliability of quantitative information, such as the overall area or the overall perimeter, in experiment 2, we presented heterogeneous elements varying in size, shape, and dimension (Rugani et al., 2013b; Rugani et al., 2015). We randomly selected heterogeneous elements among patterns of shapes (out of a pool of 10 different shapes) of up to 10 different colors and up to 10 different sizes (ranging between 0.5 cm and 2 cm in diameter/side length). Importantly, we omitted colors that are presumably highly attractive to chicks, such as red or yellow (Taylor et al., 1969; Salzen et al., 1971; Ham and Osorio, 2007; Santolin et al., 2020), to prevent a potential preference for a stimulus based solely on the presence of elements of these colors. Instead, we used shades of green, gray, blue, purple, and brown.

The rearing numerosities were: 10 (Positive stimulus, *S*_p_) and 5 (Neutral stimulus, *S*_n_) in experiments 1 and 2; 10 (*S*_p_) and 20 (*S*_n_) in experiment 3; and 20 (*S*_p_) and 10 (*S*_n_) in experiment 4; see Figure 1. At test, chicks were presented with 2 novel stimuli, composed of elements that each group experienced during rearing but arranged in a different spatial configuration. Depending on the experiment, the testing numerosities could be familiar or novel. Specifically, the testing numerosities in experiments 1 and 2 were 10 (which was the positive numerosity, *S*_p_, and its selection corresponded to the absolute strategy) and 20 (the stimulus corresponding to the relative strategy, *S*_r_). In experiments 3 and 4, the testing numerosities were 5 and 10. Numerosity 5 corresponded to the relative strategy (*S*_r_) in experiment 3, while it allowed us to control for novelty (unfamiliar stimulus, *S*_u_) in experiment 4.

We decided to use red, a highly attractive color to young chicks (Taylor et al., 1969; Salzen et al., 1971; Ham and Osorio, 2007; Santolin et al., 2020), rather than black, in experiments 3 and 4 to further encourage chicks to approach the stimuli, thus attempting to reduce the number of chicks that were excluded for remaining in the starting area for the entire duration of the test. In all experiments, we maintained the same numerical ratio (1:2) during rearing and testing.

In all cases in which the numerosity 20 was presented using homogenous elements, that is, during the test of experiment 1 as well as during rearing of experiments 3 and 4, the cardboard images of both stimuli (10 and 20) had to be enlarged to be able to maintain a side length of 1 cm and an interelement distance ranging between 0.4 cm and 2.5 cm. For the heterogenous stimuli, the dimensions remained unchanged and the minimum distance between the elements ranged from 0.2 cm to 3.5 cm.

### Test apparatus

Testing took place in an experimental room, adjacent to the rearing room, in which temperature and humidity were controlled (25 °C and 70%, respectively) and which was kept dark except for light shining from a central lamp (100 W) placed at a height of 50 cm above the apparatus. The apparatus consisted of an empty cage identical to the rearing cages, except for the insides of the cage’s walls and the floor, which were covered in uniform white opaque plastic sheets (Regolin et al., 2011). Testing stimuli were directly attached to one of the walls of the cage (side by side), centrally (4 cm from each corner) at a height of 2.5 cm above ground level. Which stimulus (*S*_p_/*S*_u_ or *S*_r_) was presented on either side was counter-balanced across subjects.

**Figure 2.**
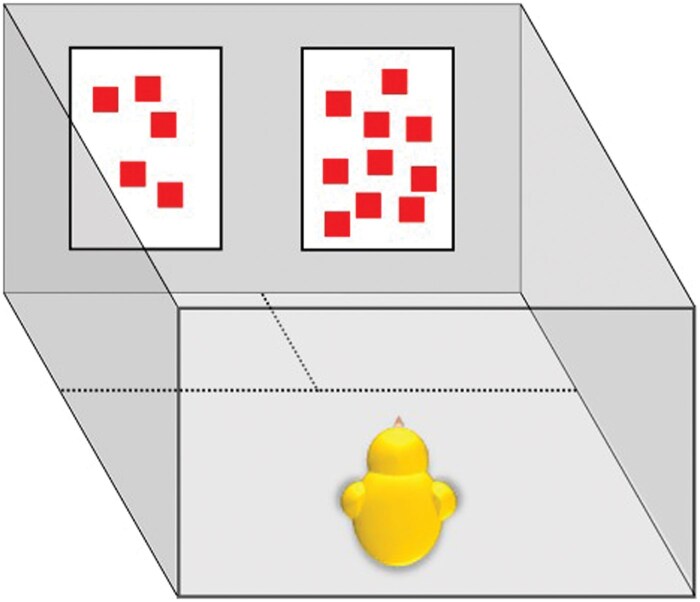
Schematic drawing of the test set-up. The stimuli presented correspond to a test situation in Experiment 3. The dashed lines indicate the borders of the preference zones.

To measure the chick’s response, pencil-drawn lines divided the cage into three areas: two small areas (14 × 16 cm) located in front of the two stimuli at the shorter end of the apparatus, and a larger section (24 × 28 cm) at the opposite end, where the chick was placed at the start of the trial, see Figure 2.

We opted for this apparatus design because it enabled chicks to simultaneously view both stimuli and move freely between them, even when in close proximity to one (Regolin et al., 2011). This overcomes limitations of a previously used runway apparatus (2013b), in which the two stimuli are positioned at opposite ends of the arena. In such a setup, chicks could be close to one stimulus while turning their back on it and viewing the other stimulus from a distance.

### Test procedure

On the morning of the third day, the testing day, each chick underwent an unrewarded test where no food was provided. At the beginning of the test, each chick was centrally placed in the starting area, with its head oriented toward the opposite wall of the apparatus where the stimuli were attached. Once the chick had been placed in the arena, the 6-min test session started, during which we recorded the chick’s behavior using a video camera positioned above the apparatus. This ensured that the chicks’ behavior was neither influenced nor disturbed by the presence of the experimenter.

### Behavior coding

Chicks’ preference was defined by their presence in one of the three areas. Time spent in each area was scored using a computerized event recorder. Chicks were considered to have chosen an area when at least ¾ of their body had crossed the demarcation line. Every time the bird entered a certain choice area, the time (s) counter for that area was set until the chick had walked out of it. No choice was scored whenever chicks were either in the starting area or exactly on the midline between 2 (or all three) areas. The overall number of seconds spent by the chick within the choice area located by the two stimuli during the whole test was considered.

For each of the 6 min, we recorded the time chicks spent in the 2 choice areas and in the starting area.

Based on these times, we calculated a preference index for each of the 6 min; for example, six indices per chick. The preference index was calculated according to the formula *T*(*S*_p_)/(*T*(*S*_p_) + *T*(*S*_r_)) (Regolin et al., 2011; Rugani et al., 2013b), with *T*(*S*_p_) being the time spent in the area with the positive stimulus and *T*(*S*_r_) being the time spent in the area with the stimulus consistent with the relative strategy, *S*_r_; in experiment 4, *T*(*S*_r_) was not present thus in the formula it was replaced by *T*(*S*_u_). Whenever a chick did not leave the starting area during one of the minutes, it was assigned a preference index of 0.5 (as otherwise the denominator would have amounted to 0). Chicks that never left the starting area during the entire 6-min test session were excluded from the study. This led to the exclusion of 20 chicks in experiment 1, 30 chicks in experiment 2, 16 in experiment 3, and 23 in experiment 4. Note that these numbers have already been subtracted from the final sample sizes presented in Figure 1.

Preference index values of around 0.5 indicated no preference for either stimulus; values > 0.5 indicated a preference for *S*_p_ (or *S*_u_ in the case of experiment 4) and values < 0.5 indicated a preference for *S*_r_.

### Statistical analyses

Statistical analyses were performed in R version 4.3.0 (R Core Team, 2023). P values below 0.05 were considered statistically significant.

To assess whether chicks exhibited a significant preference for the positive stimulus, *S*_p_ (corresponding to the absolute strategy), or for the relative stimulus, *S*_r_ (corresponding to the relative strategy), we calculated and plotted the 95% CIs of the preference index for each experiment (1, 2, 3, 4). As each chick was observed for 6 min, six preference indices were calculated for each subject. We considered the preference index to deviate significantly from 0.5 (chance level) whenever the 95%-CI did not overlap with 0.5. We repeated the same procedure for individual preference indices, using the individual preference indices calculated based on the sum of the time spent in the area close to *S*_p_ or close to *S*_r_ across all 6 min for each chick. Additionally, we checked whether chicks’ preference index changed across time. For instance, the strength of chicks’ preference for symmetrical or asymmetrical shapes, after a rearing procedure similar to ours, was shown to change with time (Mascalzoni et al., 2012). We thus included minute of the test (ranging from 1 to 6; z-transformed) in the model, see below.

To be able to calculate the CIs, we fitted a beta regression model (R package glmmTMB version 1.1.7 (Brooks et al., 2017)) with the syntax Preference_Index ∼ Group + z. Minute + (1|Subject). We set the “link” argument to “ordbeta”. For the individual preference indices, Subject was not included as a random effect, and minute was not included as a fixed effect, as the data were already summarized per subject.

For each of the two models, we subsequently computed and plotted the CIs using the functions “emmeans” and “emmip” within the “emmeans” package, version 1.8.7 (Lenth, 2022).

Moreover, to determine whether the use of physical variables, such as color, shape, and size of the elements, influenced chicks’ preference, we conducted a Mann-Whitney U-test to compare chicks’ preference indices (all trials) between experiments 1 (homogenous elements) and 2 (heterogenous elements).

#### Power analysis

Given that our sample sizes were inherently limited and unequal between the experiments due to the different numbers of chicks being excluded in each experiment, we conducted a post-hoc power analysis. For the main analysis, whether or not each experimental group’s preference index differed significantly from zero, we calculated the effect sizes based on our sample mean subtracted by 0.5 (chance level) and divided the difference by the SD of our sample. We then conducted a power test for proportion tests (pwr.p.test, R package pwr (Champely, 2020)) for each experiment. For a significance level of 0.5, power was at 0.39 for Group 1 (*n* = 40 × 6 minutes of observation), 0.07 for Group 2 (*n* = 40 × 6), 0.20 for Group 3 (*n* = 26 × 6), and 0.06 for Group 4 (*n* = 19 × 6). A low power was to be expected, given that all group mean preference indices were very close to 0.05 and, hence, effect size was very low. This might indicate that, despite low power, the nonsignificant results reflect the absence of a group-level effect rather than a false negative result. In any case, post-hoc power, calculated based on a single study should be interpreted with caution (Levine and Ensom, 2001; Zhang et al., 2019).

## Main Results

### Absolute or relative strategy?

#### Descriptive results

The mean preference index in experiment 1 (*n* = 40) was 0.54 with an SD of 0.34. Experiment 2 (*n* = 40) yielded very similar results, with mean = 0.54 and SD = 0.34. In experiment 3 (*n* = 25), chicks’ mean preference index was at 0.47 and the SD amounted to 0.34. Chicks in experiment 4 (*n* = 19) showed a mean preference index of 0.51 with an SD of 0.32. The individual preference indices in experiments 1, 2, 3, and 4 had a mean of 0.58, 0.49, 0.48, and 0.43, respectively. The corresponding SDs were 0.35 (experiment 1), 0.35 (experiment 2), 0.35 (experiment 3), and 0.31 (experiment 4).

#### Strategy preference

**Figure 3.**
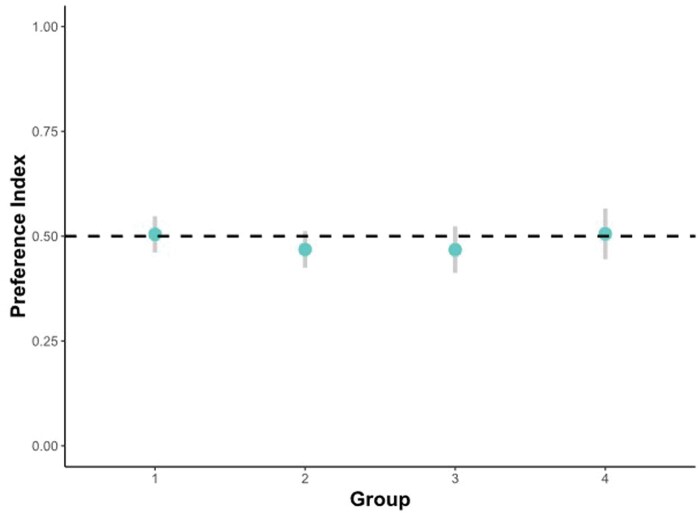
95%-CIs for chicks’ preference index across all 6 min in the 4 experiments. Experiment 1 (homogenous elements, *S*_p_ larger): [0.461; 0.548]; experiment 2 (heterogenous elements, *S*_p_ larger): [0.425; 0.512]; experiment 3 (homogenous elements, *S*_p_ smaller): [0.412; 0.524]; experiment 4 (homogenous elements, control for preference for novel stimuli): [0.445; 0.566]. Dashed line indicates a preference index of 0.5, that is, no preference.

Looking at the 95%-CIs of chicks’ preference indices in the 4 experiments, no significant deviation from 0.5, that is, no significant preference for the absolute or the relative strategy, emerged, see Figure 3. The time that passed during the test, that is, the minute of observation, did not have a significant effect on chicks’ preference index (z = −0.28, *P* = 0.78).

**Figure 4.**
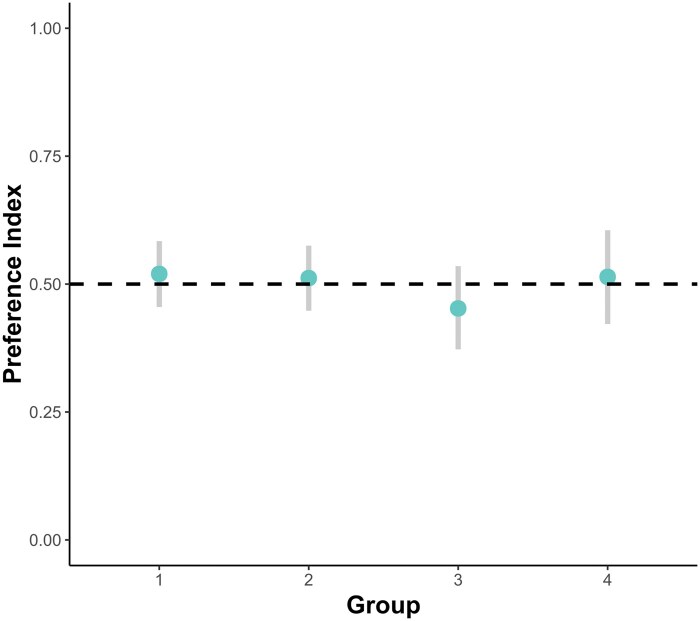
95%-CIs for chicks’ preference index per subject in the 4 experiments. Experiment 1 (homogenous elements, *S*_p_ larger): mean = 0.520, CI = [0.455; 0.584]; experiment 2 (heterogenous elements, *S*_p_ larger): mean = 0.512, [0.448; 0.575]; experiment 3 (homogenous elements, *S*_p_ smaller): mean = 0.452, CI = [0.372; 0.535]; experiment 4 (homogenous elements, control for preference for novel stimuli): mean = 0.514, CI = [0.422; 0.605]. Dashed line indicates a preference index of 0.5, that is, no preference.

Likewise, the preference indices computed per individual did not deviate significantly from 0.5 (no preference), see Figure 4.

#### Influence of physical variables

The Mann-Whitney U-test conducted to test between significant difference in preference indices between experiments 1 (homogeneous elements) and 2 (heterogenous elements) did not indicate a significant difference between the 2 experiments: *n* = 80, W = 865.5, *P* = 0.53.

### Absolute and relative strategy?

#### Statistical analyses

The analyses reported above did not reveal a preference for either an absolute or a relative discrimination strategy on a group level. Nevertheless, we were interested to know whether preferences for one or the other strategy might exist on an individual level.

For this purpose, we investigated the distribution of the individual preference indices (calculated for each subject) reported above, by plotting the density function of these indices (R package ggplot2, version 3.4.2 (Wickham, 2016)). Additionally, we tested the preference index distribution of each experiment for bimodality (i.e., for significant deviation from unimodality), using the “modetest” function within the R package “multimode” version 1.5 (Ameijeiras-Alonso et al., 2021) which is based on the Ameijeiras-Alonso excess mass test (Ameijeiras-Alonso et al., 2019). Subsequently, we employed the function “locmodes” of the same packages to localize the modes for experiments for which the modetest yielded a significant result, setting the “mod0” argument to 2.

As described above, we also aimed to explore whether chicks’ preference might be influenced by physical variables, assessed through the use of heterogeneous stimuli in experiment 2. Even though the U-test reported above did not suggest a significant difference between the preference indices in experiments 1 and 2, the distributions of the individual preference indices might nevertheless differ between the 2 experiments. To investigate whether the 2 distributions differed significantly, we conducted a Kolmogorov-Smirnov test.

#### Results

**Figure 5.**
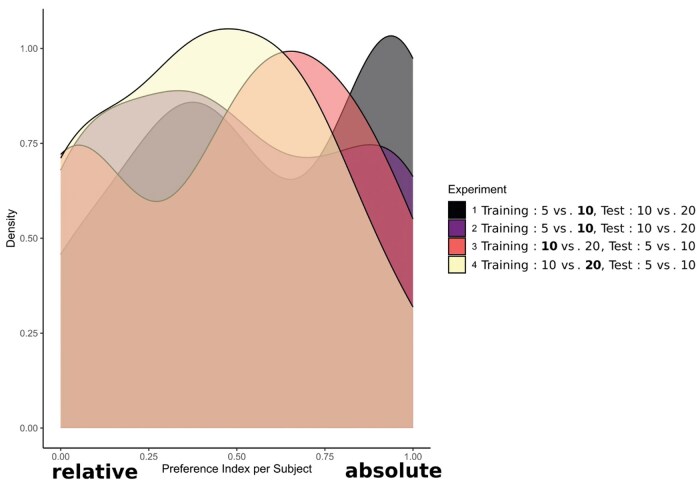
Density plot of chicks’ individual preference indices per experiment. Black: experiment 1 (Rearing: 5 vs. 10, *S*_p_ = 10, Test: 10 vs. 20, homogenous stimuli); Purple: experiment 2 (Rearing: 5 vs. 10, *S*_p_ = 10, Test: 10 vs. 20, heterogenous stimuli); Red: experiment 3 (Rearing: 10 vs. 20, *S*_p_ = 10, Test: 5 vs. 10, homogenous stimuli); Yellow: experiment 4 (Rearing: 10 vs. 20, *S*_p_ = 20, Test: 5 vs. 10, homogenous stimuli).

Results of all experiments, except the control experiment 4, show a bimodal distribution of the individual preference indices, Figure 5. In other words, 2 subgroups of chicks seem to emerge for each experiment, one that pursues the absolute strategy (preference index > 0.5) and one that follows the relative strategy (preference index < 0.5). This impression was confirmed by the mode tests, which suggested that the preference indices in experiments 1 (*n* = 40, excess mass = 0.143, *P* = 0.032), 2 (*n* = 40, excess mass = 0.161, *P* = 0.014), and 3 (*n* = 25, excess mass = 0.230, *P* < 0.001), but not 4 (*n* = 19, excess mass = 0.174, *P* = 0.132), deviated significantly from a unimodal distribution. Based on Figure 5, this deviation from unimodality can be construed as the distributions being bimodal. The modes of the preference index in experiment 1 were estimated to be at 0.373 and 0.958, with the antimode being located at 0.683. For the preference index in experiment 2, the modes were at 0.367 and 0.930, while the antimode was estimated to be at 0.673. The modes of the preference index in experiment 3 were located at 0.023 and 0.555, and the antimode was estimated to be at 0.279. Moreover, the Kolmogorov-Smirnov test suggested that the distribution of the individual preference indices did not differ significantly between experiments 1 and 2 (*n* = 80, D = 0.175, *P* = 0.561).

## Discussion

We set out to investigate whether domestic chicks, like humans (Miletto Petrazzini et al., 2016), fish (Miletto Petrazzini et al., 2015; Miletto Petrazzini et al., 2016) and bees (Bortot et al., 2019), consistently adopt either an absolute or a relative strategy in a numerical discrimination task in which the 2 strategies conflict. At a group level, chicks exhibited no preference for either strategy, irrespective of 1) the reliability and availability of physical variables and 2) whether the smaller or larger numerosity had been associated with food during rearing.

Remarkably, chicks might show distinct individual preferences. The distribution of individual preference indices revealed two subgroups, one employing the absolute strategy and the other the relative strategy, across all experiments except the control. More precisely, the distribution of individual preference indices significantly deviated from unimodality in experiment 1, where chicks associated the larger number with food, in experiment 2, where the larger number was again rewarded and heterogeneous stimuli reduced reliance on physical cues, and in experiment 3, where the smaller number was associated with food. However, in experiment 4, where the reinforced numerosity was 20 and the test involved 5 vs. 10, a bimodal pattern did not emerge. Despite the smaller sample size in Experiment 4, the distribution of preference indices suggests that chicks’ behavior tends to emerge specifically in contexts requiring a choice between 2 distinct strategies, rather than being driven merely by the presence of two stimuli and respective preference zones. Moreover, neither the means nor the distributions of the preference indices differed significantly between experiments 1 (homogeneous elements) and 2 (heterogeneous elements), indicating that physical variables, such as color, shape, and size, did not influence chicks’ strategy preferences.

Despite these unexpected results, it is unlikely that our experimental procedure simply failed to induce and later reveal existing group-level strategy preferences in chicks. Instead, previous studies have demonstrated that paradigms similar to those applied here are effective in establishing measurable preferences. For example, chicks learned to associate food with symmetrical or asymmetrical shapes (Mascalzoni et al., 2012), small or large numerosities represented by different configurations of various shapes (Rugani et al., 2013b; Rugani et al., 2014b), and with relatively larger or smaller circles (Rosa Salva et al., 2013). Also in the present research, this paradigm seemed to succeed in inducing learning in chicks, as they did exhibit a preference, albeit only on an individual level. Future studies could include a retention test using the same stimuli presented during exposure, to assess whether chicks have formed a positive association with the reinforced stimulus. In the present study, however, no retention test was conducted, as our protocol did not include reinforcement. Under such conditions, administering a retention test before the main test may have reduced chicks’ motivation to approach either stimulus when subsequently tested with novel stimuli. The unequal sample sizes across experiments are consistent with previous studies (Regolin et al., 2011; Rosa Salva et al., 2013) and reflect a careful balance between experimental requirements (e.g., minimum sample size) and practical constraints (e.g., variability in hatching rates and subject exclusion due to nonresponsiveness).

The present study leaves open the question of whether the observed individual strategy preferences remain consistent over time, offering an avenue for future research. As in previous studies assessing preferences established during rearing (Regolin et al., 2011; Rugani et al., 2013b), we tested chicks only in a single trial of 6 min. This approach leaves open the possibility of exploring whether chicks would exhibit consistent individual preferences across multiple trials or with varying numerosities. It would be informative for future studies to retest chicks in the same task across multiple trials, in order to evaluate the stability or flexibility of individual preferences. This would not only strengthen the evidence for divergent individual strategy preferences but also provide valuable insight into their underlying nature.

Our findings, which indicate that chicks do show a preference only at the individual level, but not at the group level, contrast with the results observed in humans, fish, and bees, which demonstrated group-level preferences. This divergence could be the result of slight differences in experimental procedures. First, the variation in the numerosity employed, ranging between small and large magnitudes, could potentially explain the observed differences. Angelfish (Miletto Petrazzini et al., 2016), humans (Miletto Petrazzini et al., 2016) and our chicks were tested with the numerosity pairs 5 vs. 10 and 10 vs. 20. Similarly, guppies (Miletto Petrazzini et al., 2015) were tested with relatively large numerosities (3, 6, 12, and 24), while bees (Bortot et al., 2019) were tested with small numerosities (2 vs. 3 and 3 vs. 4). A possible difference in strategy use could be attributed to variations in the processing of small and large numerosities (Rugani et al., 2013a; Rugani et al., 2014b). However, this does not explain why chicks fail to show a preference for a relative strategy at the group level, unlike humans and angelfish tested with the same numerosities.

Second, bees, fish, and humans completed a training procedure consisting of distinct two-way choice trials. In contrast, our chicks did not undergo a supervised training procedure. Instead, they were exposed to the stimuli during rearing and were thereby able to form an association with food (Rugani et al., 2013b; Rugani et al., 2014b). Moreover, in our test, we applied a more ecological approach allowing chicks to freely explore both stimuli while measuring the time spent near the test stimuli during a single long trial. Importantly, such a free-ranging setting could additionally enhance chicks’ motivation to explore their environment, leading to more interindividual variability. It would be worthwhile to test other species using the same methodology to determine whether the divergent preferences truly reflect species and/or age differences or merely differences in experimental context. Testing the same species under different training conditions, including supervised and free-ranging trainings, could unveil whether different training can induce the use of different strategies. This could help disentangle whether the divergent preferences are driven by inherent species or age differences, or merely by differences in experimental context.

Third, another crucial difference could lie in the age of the subjects. Our study was the only one to involve juvenile animals, whereas humans, fish, and bees were tested as adults. It is plausible that interindividual variability is more pronounced at early stages of ontogeny. Therefore, a group-level preference for either the absolute or relative discrimination strategy in domestic chicks may emerge later in ontogeny. Flexibility in strategy use during early development could be advantageous, enhancing adaptability. Experience and interaction with environmental demands might subsequently shape the consolidation of either an absolute or relative strategy. Testing older domestic chickens in future studies would be informative to assess whether these individual preferences develop into a more stable, population-level bias with age and experience.

The absence of a group-level strategy preference in young animals, together with pronounced interindividual variability in numerical discrimination strategy in early life might hold similar evolutionary advantages as variability in foraging or antipredator strategies. That is, the presence of multiple distinct antipredator or foraging strategies within a population carries evolutionary advantages as it provides robustness against environmental changes (Benton, 2012), for example, climate change (Cockrem, 2022). For example, individual differences in predator-avoidance strategies and vigilance are associated with differential survival rates in red shanks (Sansom et al., 2009) and bank voles’ personality explains individual differences in foraging and predator avoidance (Mazza et al., 2019). Similarly, within small groups of ducklings, one or few individuals take on the role of the “initiator” whom others follow to bathing ponds, facilitating collective decisions while maintaining group coherence (Liste et al., 2014). Analogously, also the variability in chicks’ individual preferences in numerical discrimination might allow the population to adjust more flexibly to varying environmental challenges. In naturalistic settings, where chicks must rapidly evaluate and select between food sources, social companions or predators, having access to multiple cognitive strategies might confer a selective advantage.

Intriguingly, such variability in individual strategies, as seen here for numerical discrimination, is reflected also in chicks’ performance in geometrical middle-identification tasks. More precisely, when trained to find food in the center of an arena and then tested in an enlarged arena, chicks showed a bimodal search pattern: some chicks seemed to rely on the absolute distance from one side of the arena, while others searched in the new geometrical center by equalizing the distances from all sides (Tommasi et al., 1997; Tommasi and Vallortigara, 2000; Della Chiesa et al., 2006). These two strategies could be interpreted as remembering absolute information (absolute distance) or as learning a relative rule (equalize the distances to all sides to find the center), similar to the two numerical discrimination strategies described here. More detailed investigations have revealed that, in species like rats and humans, subjects’ strategy choice is easily influenced by the exact methodology applied (Tommasi and Thinus-Blanc, 2004; Tommasi and Giuliano, 2014). Consequently, individual strategies and their variability might be sensitive to details of the learning and testing procedure also in discrimination tasks.

In conclusion, chicks do not exhibit a preference for a relative or an absolute numerical discrimination strategy on a group level. Rather, there seem to be two distinct subgroups of chicks, which individually prefer the relative or the absolute strategy, respectively. These results are in contrast with the clear group-level preferences in bees (absolute) (Bortot et al., 2019), fish (relative) (Miletto Petrazzini et al., 2015; Miletto Petrazzini et al., 2016), and humans (relative) (Miletto Petrazzini et al., 2016) demonstrated in previous studies. This might suggest that the strategies used to solve numerical discrimination tasks vary even among vertebrates, for instance between birds, fish, and mammals. However, future studies should clarify whether these apparent differences and the interindividual variability can indeed be attributed to the species’ distinct ecologies and evolutionary histories, or rather to subjects’ age and unequal experimental procedures.

## Data Availability

Data and analysis scripts are available here: https://researchdata.cab.unipd.it/1639/

## Abbreviations:

*S*_p_: positive stimulus, that is, numerosity reinforced during rearing
*S*_n_: neutral stimulus, that is, numerosity present during rearing but never associated with food, and not corresponding to neither the relative nor the absolute strategy
*S*_r_: relative stimulus, that is, numerositiy that would be the correct choice according to the relative strategy (the larger or the smaller numerosity)
*S*_u_: unfamiliar stimulus, that is, numerosity presented in the test that is not correct for either strategy
